# Clinical features and therapeutic management of patients admitted to Italian acute hospital psychiatric units: the PERSEO (psychiatric emergency study and epidemiology) survey

**DOI:** 10.1186/1744-859X-6-29

**Published:** 2007-11-05

**Authors:** Andrea Ballerini, Roberto M Boccalon, Giancarlo Boncompagni, Massimo Casacchia, Francesco Margari, Lina Minervini, Roberto Righi, Federico Russo, Andrea Salteri, Sonia Frediani, Andrea Rossi, Marco Scatigna, the PERSEO study group

**Affiliations:** 1 Servizio Psichiatrico Diagnosi e Cura, Santa Maria Nuova Hospital, Firenze, Italy; 2 Servizio Psichiatrico Diagnosi e Cura, Sant' Anna Hospital, Ferrara, Italy; 3 Servizio Psichiatrico Diagnosi e Cura, S. Orsola Malpighi Hospital, Bologna, Italy; 4 Clinica Psichiatrica, San Salvatore Hospital, L'Aquila, Italy; 5 Istituto di Clinica Psichiatrica, Policlinico Consorziale Hospital, Bari, Italy; 6 Dipartimento di Salute Mentale, Azienda USL 16 Hospital, Padova, Italy; 7 Servizio Psichiatrico Diagnosi e Cura, Hospital of Adria, Rovigo, Italy; 8 Servizio Psichiatrico Diagnosi e Cura, Nuovo Regina Margherita Hospital, Roma, Italy; 9 Servizio Psichiatrico Diagnosi e Cura, Vimercate Civil Hospital, Milano, Italy; 10 Medical Department, Eli Lilly Italia, Firenze, Italy; 11 See Appendix for details of participating groups

## Abstract

**Background:**

The PERSEO study (psychiatric emergency study and epidemiology) is a naturalistic, observational clinical survey in Italian acute hospital psychiatric units, called SPDCs (Servizio Psichiatrico Diagnosi e Cura; in English, the psychiatric service for diagnosis and management). The aims of this paper are: (i) to describe the epidemiological and clinical characteristics of patients, including sociodemographic features, risk factors, life habits and psychiatric diagnoses; and (ii) to assess the clinical management, subjective wellbeing and attitudes toward medications.

**Methods:**

A total of 62 SPDCs distributed throughout Italy participated in the study and 2521 patients were enrolled over the 5-month study period.

**Results:**

Almost half of patients (46%) showed an aggressive behaviour at admission to ward, but they engaged more commonly in verbal aggression (38%), than in aggression toward other people (20%). A total of 78% of patients had a psychiatric diagnosis at admission, most frequently schizophrenia (36%), followed by depression (16%) and personality disorders (14%), and no relevant changes in the diagnoses pattern were observed during hospital stay. Benzodiazepines were the most commonly prescribed drugs, regardless of diagnosis, at all time points. Overall, up to 83% of patients were treated with neuroleptic drugs and up to 27% received more than one neuroleptic either during hospital stay or at discharge. Atypical and conventional antipsychotics were equally prescribed for schizophrenia (59 vs 65% during stay and 59 vs 60% at discharge), while atypical drugs were preferred in schizoaffective psychoses (72 vs 49% during stay and 70 vs 46% at discharge) and depression (41 vs 32% during stay and 44 vs 25% at discharge). Atypical neuroleptics were slightly preferred to conventional ones at hospital discharge (52 vs 44%). Polypharmacy was in general widely used. Patient attitudes toward medications were on average positive and self-reported compliance increased during hospital stay.

**Conclusion:**

Results confirm the widespread use of antipsychotics and the increasing trend in atypical drugs prescription, in both psychiatric in- and outpatients.

## Background

Several countries, mainly in North America and Europe, have adopted psychiatric units into general hospitals, as an alternative to the classic psychiatric hospital, for referral of acute patients with mental illnesses. In Italy, since 1978, the law prescribes that psychiatric patients can only be admitted to hospitals through these specific emergency structures, called SPDCs (Servizio Psichiatrico Diagnosi e Cura, i.e. psychiatric service for diagnosis and management). The implementation of this mental health reform law shifted the focus of care from mental hospitals to community services. Since their institution, patients remain in SPDCs only during the acute phase of their illness. At discharge, they usually receive therapeutic prescriptions and are no longer followed by SPDC structures, but by territorial services (some of which are specialized on, e.g., drug addiction, etc), which are not part of the general hospital system [1]. SPDCs should be the perfect setting for studying psychiatric patients at their hospital presentation, however bed shortages, emphasis on acuity and a continuous emergency situation render it rather difficult to implement clinical research and epidemiology programs.

In order to study, from an epidemiological perspective, the Italian population referring to psychiatric emergency structures, the PERSEO project – for psychiatric emergency study and epidemiology – was designed. The whole project consisted of two phases: a pilot phase involving 15 SPDCs, performed in 2002, called the EPICA study (for epidemiology in psychiatry: acute cases), which collected preliminary epidemiological information and validated in Italian the Modified Overt Aggression Scale (MOAS) and the Nurses' Observational Scale for Inpatient Evaluation (NOISIE) [2]. The second phase consists of the PERSEO study, a larger observational multi-centre study involving 62 Italian SPDCs, aimed at assessing the sociodemographic and clinical characteristics of patients, their pathways to psychiatric admission, and describing their behaviour, subjective wellbeing, management and attitude towards treatment during SPDC stay. Study characteristics and methods have been described in depth in a previous paper [3]. We were also interested in evaluating the possible differences between first admission versus repeated admission patients, also referring to data specifically concerning first admission patients and their aggressive behaviour, which have recently been the object of a specific publication [4]. The present paper focuses mainly on the management of SPDC patients and their attitudes towards pharmacological treatments.

## Methods

PERSEO was designed as a cross-sectional observational multi-centre study aimed at assessing some epidemiological features of patients referring to Italian SPDCs, and in particular their overall management and psychopharmacological treatment in the emergency setting. A total of 62 SPDCs, distributed throughout Italy, participated in the study. Following approval by the Ethics Committees of the participant institutions and obtainment of the patients' written informed consent, all consecutive subjects aged 18 years or more admitted to an SPDC between September 2003 and April 2004 were enrolled into the study. All patients were admitted to the study only once over the enrolment period. The subjects were evaluated at admission, and then daily for the first 3 days of hospital stay and at discharge or at day 30, whichever came first.

Psychiatric symptoms were evaluated by the 24 items Brief Psychiatric Rating Scale (BPRS) [5] and the Brief Symptoms Inventory (BSI) [6]. The patients' subjective wellbeing was assessed by the Subjective Wellbeing under Neuroleptics (SWN) scale [7], while the Drug Attitude Inventory (DAI-30) [8] was used to measure their attitude towards the pharmacological treatments. The DAI-30 is a relatively widely used self-report inventory that focuses on the subjective effect of antipsychotic medications. To assess the prevalence of aggressive behaviours, the Modified Overt Aggression Scale (MOAS) [9], which is the modified version of the Overt Aggression Scale (OAS), developed by Yudofski et al [10] and recently validated in Italian by Margari et al [2], was used.

Sociodemographic and anamnestic data, life habits, risk factors for psychiatric disease, reason for hospital admission, referring structure, clinical and psychometric evaluations, concomitant diseases, previous and ongoing psychiatric treatment, admission and discharge diagnoses, and treatments administered in SPDC were recorded on case record forms (CRF). For a full statistical analysis, patient diagnoses were grouped as described in Table 1.

**Table 1:** Diagnosis grouping by ICD9-CM code

**Diagnosis group**	**ICD9-CM CODES**
Schizophrenia	295/295.xx (not 295.7) schizophrenia
Paranoid status and other non-organic psychoses	297/297.xx Paranoid status
	298.2 Reactive confusional status
	298.3 Acute paranoid reaction (delirious bouffee)
	298.4 Psychoenic paranoid psic.
	298.8 Other reactive psychoses
	298.9 SAI psychoses
Affective psychosis, manic episodes, excitement status	296.0x Single ep. mania
	296.1x Recurrent ep. mania
	296.4x Affective bipolar sind. manic ep.
	296.81 Atypical manic sind
	296.6x Affective bipolar sind. mixed ep.
	298.1 Agitataed type psy.; psychogen. excitat.
Affective psychosis, depression,	296.2 Depr. single ep.
depressive status	
	296.3x Depr. recurrent ep.
	296.5x Bipolar affective sind. depr. ep.
	296.82 Atypical depression
	298.0 Atyp. psychosis depressive type
	311.x Depression not other class.
	296.7x Manic-depr. sind circular type SAI
	296.8x (except .81 o .82) Manic depr sind SAI
	296.9x Affective psy. SAI
Schizoaffective psychosis	295.7
Personality disorders	301/301.xx
Neurotic disorders	300/300.xx
Acute stress reactions, adaptation reactions	308/308.xx/309/309.xx
Substance abuse, dependence	303/303.xx/304/304.xx/305/305.xx
	291/291.xx/292/292.xx
Dementia and psycho-organic	290/290.xx dementia
syndromes	
	293.x Transient organic psychoses
	294/294.xx Chronic organic psychoses
	310/310.xx Frontal lobe synd. And other non-
	psychotic from brain damage
Mental retardation, infantile psychoses	314/314.xx/Infant hypercinetc sindr.
	315/315.xx/Specific devlopm. retardation
	317/317.xx/Mental retardation
	318/318.xx/
	319/319.xx/
	299/299.xx Infant psychoses, autism
Others	302.x Sexual deviations and disturb.
	306.x Physical disfunctions with psych. origin
	307.xx (except 307.1, 307.5)
	307.1 anorexia
	307.5x other alimentary disturb.
	312/312.xx/
	313/313.xx/
	316

Project and data management, and statistics were conducted by MediData Studi e Ricerche. Data were analyzed using SAS for Windows, release 8.2 (The SAS Institute Inc.). All quantitative variables were described by means, standard deviations and ranges. Absolute and relative frequency distributions were given for qualitative variables. Comparisons were performed by Student's *t *test for mean values, chi-square test, or Fisher exact test. When multiple comparisons were performed, Bonferroni's correction was taken into account. More details on the methodology of the study can be found in Ballerini et al 2005 [4].

## Results

Overall, 2521 patients were enrolled in the 62 participating SPDCs, with a consequent guarantee of a generous geographic coverage of the country. As 49 patients were not viable due to protocol violations or missing data, the analyses were conducted on 2472 patients (98.1%). Their sociodemographic characteristics are summarized in Table 2. Mean age was significantly higher among women than men (p < 0.001). The percentage of smokers and alcohol or drug abusers was significantly higher in men (p < 0.001). Admission was voluntary in 85% of cases, involuntary in the remaining 15% of cases; a significantly higher percentage of women than men were voluntary admitted (p < 0.001). In addition, marital and occupational status results were distributed in a statistically different manner between males and females (p < 0.001).

**Table 2:** Sociodemographic characteristics of valuable patients (n = 2472)

**Characteristic**	**Parameter**		
		**Females**	**Males**

**Gender, n (%)**		1214 (49.1)	1258 (50.9)

	**Mean (SD)**	**Females**	**Males**

**Age**	43.7 (14.2)	45.7 (14.3)*	41.7 (13.8)*

**Occupational status, n (%)**	**Overall**	Females*	Males*

Employed	543 (22.0)	225 (18.5)	318 (25.3)
Housewife	328 (13.3)	325 (26.8)	3 (0.2)
Student	47 (1.9)	19 (1.6)	28 (2.2)
Unemployed	658 (26.6)	249 (20.5)	409 (32.5)
Retired	703 (28.4)	309 (25.5)	394 (31.3)
Disabled	103 (4.2)	40 (3.3)	63 (5.0)
Other	42 (1.7)	21 (1.7)	21 (1.7)
NA	48 (1.9)	26 (2.1)	22 (1.7)

**Marital status, n (%)**	**Overall**	Females*	Males*

Single	1079 (43.6)	385 (31.7)	694 (55.2)
Married	564 (22.8)	365 (30.1)	199 (15.8)
Widow	103 (4.2)	83 (6.8)	20 (1.6)
Divorced	271 (10.9)	157 (13.0)	114 (9.0)
NA	455 (18.4)	224 (18.4)	231 (18.4)

**Life habits, n (%)**	**Overall**	Females*	Males*

Smokers	1421 (57.5)	542 (44.6)	879 (69.9)
Alcohol abusers	462 (18.7)	149 (12.3)	313 (24.9)
Drug abusers	86 (3.5)	15 (1.24)	71 (5.6)

**Admission status, n (%)**	**Overall**	Females*	Males*

Voluntary	2103 (85.1)	1073 (88.4)	1030 (81.9)
Involuntary	364 (14.7)	140 (11.5)	224 (17.8)
NA	5 (0.2)	1 (0.1)	4 (0.3)

*Males versus females, p < 0.001; NA: Not available/unknown.

A total of 772 (31.2%) patients were referred by specialized structures, either private psychiatrists or public mental health centres, 362 (14.6%) by non-specialized health professionals (general practitioners or physicians operating in emergency structures), 340 (13.8%) by other hospitals or rehabilitation structures, while the majority of patients (998; 40.4%) had not contacted a physician before admission to an SPDC. Among the reasons for admission, poor compliance to psychopharmacological treatment was reported in 44.9% of the overall study population and in 52.2% of subjects presenting with severe psychiatric symptoms.

A total of 542 (21.9%) patients had no diagnosis at admission. The diagnoses of the remaining 1930 patients (78.1%) are summarized in Table 3 (lefthand column). At discharge from the SPDC, a psychiatric diagnosis had been established in almost all patients (n = 2407; 97.4%). The distribution of diagnoses at discharge results were higher in women than in men for affective diseases and neurotic disorders, while schizophrenia and substance abuse were more often diagnosed in men than women (Table 3, righthand columns).

**Table 3:** Diagnoses at admission to SPDC and at discharge. The percentage is calculated on patients with an established diagnosis at admission and at discharge.

**Diagnosis**	**Admission n (%)**	**Discharge n (%)**		
		**Overall**	**Females**	**Male**s

Schizophrenia, paranoid status, other non-organic psychosis	698 (36.2)	843 (35.0)	342 (28.9)*	501 (41.0)*
Affective psychosis, depression, depressive status	315 (16.3)	401 (16.7)	246 (20.8)*	155 (12.7)*
Personality disorders	268 (13.9)	325 (13.5)	174 (14.7)	151 (12.4)
Affective psychosis, manic episodes, excitement status	176 (9.1)	213 (8.9)	121 (10.2)†	92 (7.5)†
Schizoaffective psychosis	138 (7.2)	164 (6.8)	93 (7.9)†	71 (5.8)†
Substance abuse, dependence	94 (4.9)	135 (5.6)	32 (2.7)*	103 (8.4)*
Neurotic disorders	77 (4.0)	103 (4.3)	69 (5.8)*	34 (2.8)*
Dementia and psycho-organic syndromes	43 (2.2)	78 (3.2)	36 (3.0)	42 (3.4)
Mental retardation, infantile psychoses	29 (1.5)	28 (1.2)	9 (0.8)	19 (1.6)
Acute stress reactions, adaptation reactions	12 (0.6)	47 (2.0)	28 (2.4)	19 (1.6)
Others/NA	80 (4.1)	70 (2.9)	34 (2.9)	36 (2.9)

**Total no of patients with diagnosis **(% of total PERSEO population)	1930 (78.1)	2407 (97.4)	1184 (97.5)	1223 (97.2)

*Males versus females, p < 0.001; †males versus females, p < 0.05.

In total, 94% of patients (n = 2325) were viable for the MOAS and almost half of them (46.4%) had a MOAS score greater than 0 at admission. The aggressive behaviour recorded was most commonly a verbal one (37.8%), while aggression against other people accounted for 20.5% of patients and aggression against property for 18.3%. Auto-aggression episodes occurred in 15.7% of cases.

At admission to an SPDC, 66.3% of patients had received a previous psychopharmacological treatment, as summarized in Table 4 (lefthand column), most frequently (73.1%) a combination therapy; the most common combinations were antidepressants with benzodiazepines (7.4%) and conventional antipsychotics with benzodiazepines (6.2%). Single treatments were 9.0%, and 6.8% conventional and atypical antipsychotics, 4.9% benzodiazepines, 3.4% antidepressants, and 2.8% mood stabilizers. A psychoactive medication was administered during SPDC stay to 98.3% of patients and prescribed at discharge to 95.4% (Table 4, middle and right columns).

**Table 4:** Psychopharmacological treatment before admission to SPDC, during SPDC stay and at discharge. Percentages are calculated on patients under treatment at admission, during hospital stay and at discharge.

**Type of drug**	**At admission n (%)**			**In SPDC n (%)**			**At discharge n (%)**		
	
	**Overall**	**Females**	**Males**	**Overall**	**Females**	**Males**	**Overall**	**Females**	**Males**
Benzodiazepine	960 (58.6)	513 (42.3)*	447 (35.5)*	1927 (79.3)	964 (79.4)	963 (76.6)	1604 (68.0)	795 (65.5)	809 (64.3)
Atypical antipsychotic	700 (42.7)	321 (26.4)†	379 (30.1)†	1215 (50.0)	571 (47.0)†	644 (51.2)†	1222 (51.8)	577 (47.5)	645 (51.3)
Conventional antipsychotic	690 (42.1)	316 (26.6)†	374 (29.7)†	1268 (52.2)	575 (47.4)*	693 (55.1)*	1046 (44.4)	483 (39.8)†	563 (44.8)†
Antidepressant	591 (36.1)	361 (29.7)*	230 (18.3)*	808 (33.3)	476 (39.2)*	332 (26.4)*	795 (33.7)	464 (38.2)*	331 (26.3)*
Mood stabilizer	509 (31.1)	263 (21.7)	246 (19.6)	764 (31.5)	391 (32.2)	373 (29.7)	774 (32.8)	392 (32.3)	382 (30.4)
Anticholinergic	131 (8.0)	53 (4.4)†	78 (6.2)†	301 (12.4)	119 (9.8)*	182 (14.5)*	261 (11.1)	107 (8.8)§S	154 (12.2)§S
Other	67 (4.1)	28 (2.3)†	15 (1.2)†	215 (8.9)	92 (7.6)*	55 (4.4)*	101 (4.2)	43 (3.5)†	24 (1.9)†
	
**Total no. of patients on treatment**	1638 (66.3)	825 (68.0)	813 (64.6)	2429 (98.3)	1197 (98.6)	1232 (97.8)	2358 (95.4)	1161 (95.6)	1197 (95.2)
(% of total PERSEO population)									

*Males versus females, p < 0.001; †males versus females, p < 0.05; §males versus females, p < 0.01

Consistent with psychiatric diagnoses, antidepressants are more frequently prescribed for women than men, while antipsychotics and anticholinergics are more frequently prescribed for men at any time of data collection. Benzodiazepines were more frequently prescribed to women before SPDC admission but, during hospitalization and at discharge, this difference reduced to a non-significant level.

**Figure 1:**
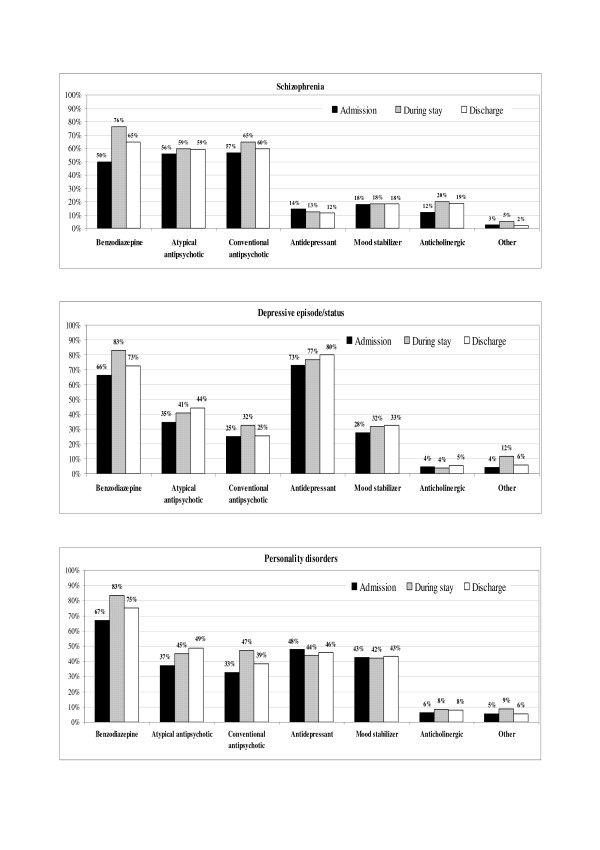
**Psychopharmacological therapies prescribed at admission, during stay and at discharge from SPDC for main (n = 100) psychiatric diagnoses (schizophrenia, depressive episode/status, personality disorders)**. Percentages are calculated on patients with at least one psychoactive drug prescribed within each diagnostic group. A patient could be taking more than one drug at the same time. Schizophrenia includes paranoid status and other non-organic psychoses.

**Figure 2:**
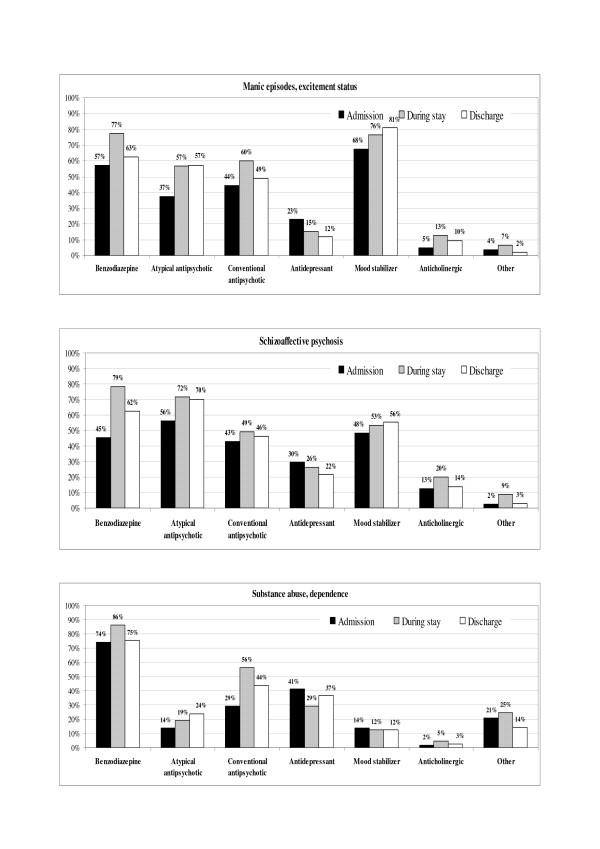
**Psychopharmacological therapies prescribed at admission, during stay and at discharge from SPDC for main (n = 100) psychiatric diagnoses (manic episodes/excitement status, schizoaffective psychosis, substance abuse/dependence)**. Percentages are calculated on patients with at least one psychoactive drug prescribed within each diagnostic group. A patient could be taking more than one drug at the same time. Schizophrenia includes paranoid status and other non-organic psychoses.

The psychopharmacological treatments prescribed before admission to the SPDC, during hospital stay and at discharge, stratified per psychiatric diagnosis groups, are reported in Figures 1 and 2.

The switch rates for neuroleptic therapy were analysed. Overall, 49% of patients were taking neuroleptic drugs (either typical or atypical) and 7% received more than one neuroleptic, before admission to hospital. After admission to an SPDC, 83% were prescribed neuroleptic drugs and 27% had a combination of them. The prescription of atypical antipsychotics increased during SPDC stay for all diagnostic groups, slightly for schizophrenic patients (+7%), but much more markedly for manic (+52%) and schizoaffective (+28%) patients. In addition, conventional antipsychotic prescriptions increased in all diagnostic groups during hospital stay, though with a slightly different pattern: from +14% for schizophrenia up to +45% for personality disorders. In 74.3% of the patients who were taking a combination of conventional and atypical antipsychotics at admission, the combination was confirmed during hospital stay and/or at discharge. Similarly, 64.5% and 60.6% of the patients who were taking only atypical and only conventional antipsychotics, respectively, kept on with the same treatment during hospital stay and/or at discharge. Patients who were not under neuroleptics at admission started antipsychotic therapy during hospital stay and/or at discharge, with atypical only, conventional only, both conventional and atypical drugs in 22.2%, 28.4%, and 20.4% of cases, respectively. As shown in Figures 1 and 2, at discharge, atypical antipsychotics prescriptions were in general confirmed, while conventional antipsychotics tended to be reduced.

In general, the patients showed improvements from admission to discharge in their psychotic symptoms, as evaluated both by psychiatrists (by BPRS) and by the patients themselves (by BSI). Subjective wellbeing was evaluated on the SWN scale by 793 patients, with both admission and discharge questionnaires fully compiled. SWN was rated by all patients, either with or without antipsychotics. SWN mean total score increased from admission to discharge in all antipsychotic treatment groups: atypical only, conventional only, both conventional and atypical (Table 5). Overall, the patients' attitude towards neuroleptics was positive, as indicated by the high percentage of patients with a positive DAI-30 total mean score in all treatment groups at admission, which further increased at discharge (Table 5). At admission 44% of subjects were receiving psychological support or had received it within 1 month prior to admission. Psychological support was then administered to 65% of patients during hospital stay and prescribed at discharge to approximately 68% of patients.

**Table 5:** Mean score (± SD) at the subjective wellbeing (SWN) scale and number (%) of patients with positive DAI-30 score at admission to and discharge from SPDC, in patients treated with antipsychotics (atypical, conventional or both)

**Rating scale**	**Atypical group**	**Conventional group**	**Mixed group**
SWN			
Admission	73.1 (17.4)	76.0 (19.0)	74.6 (18.2)
Discharge	79.8 (16.4)	83.4 (15.9)	84.5 (14.9)
DAI-30 > 0			
Admission	249 (68.8)	212 (64.2)	169 (67.6)
Discharge	292 (80.7)	259 (78.5)	197 (78.8)

SWN: possible scores range from 20 to 120, with higher scores indicating greater wellbeing. DAI-30: a score > 0 indicates a positive attitude toward pharmacological treatments.

## Discussion

PERSEO is a naturalistic, observational study aimed at assessing the epidemiological and clinical characteristics of patients admitted to Italian psychiatric units, including sociodemographic features and life habits, diagnoses, behaviours, and psychiatric symptoms at presentation, as well as their clinical management, subjective wellbeing and attitudes toward medications. To our knowledge, with 62 SPDC involved and 2521 cases enrolled, the PERSEO study represents the largest epidemiological survey performed in recent years on patients admitted to Italian psychiatric emergency structures.

Males and females were equally represented in our study population, but a statistically significant difference was observed between genders for all of the demographic characteristics (age, life habits, marital and occupational status), with females being on average 4 years older than males. This seems to be consistent with the delayed onset of psychosis in females as compared to males previously reported by several authors, in particular for schizophrenia, which has been explained by some authors by the role played by estrogens in modulating serotoninergic function [11-13]. The percentage of smokers was quite high in our patient population (57.5%), but this was expected, as it is well known that psychiatric patients are more vulnerable to nicotine-dependence and rates of smoking are about two- to fourfold higher in patients with psychiatric disorders [14-16]. Conversely, the percentage of drug abusers was quite low, even though it has been reported that the prevalence of comorbidity of psychosis and substance abuse has been rising during the last 10–20 years and substance use disorders are overrepresented in subjects with schizophrenia and bipolar and bipolar spectrum disorders [17-19]. Consistent with literature reports [20-22], the prevalence of smokers, drug or alcohol abusers in males was statistically higher than in females. Voluntary admissions to SPDCs were statistically higher for females than males.

Both at admission to SPDC and at discharge, the most frequent diagnosis was schizophrenia, followed by depression and personality disorders; interestingly, there were no relevant changes in diagnosis during hospital stay, indicating that patients' mental disorders had already been well recognized before unit admission. However, during SPDC stay psychiatric diagnoses were also defined for almost all patients admitted without diagnosis, with 97.4% of the overall patient population diagnosed at discharge.

It has been known for some time that psychiatric patients, in particular those affected with schizophrenia, are likely to engage in acts of aggression [23-25], but the importance of distinguishing among different types of aggression has been recently underlined [26]. Many of our patients showed some type of aggressive behaviour (46%), but they engaged in verbal aggressive episodes more commonly (38%) than in aggression against other people (21%). As per diagnosis, the most frequent diagnosis among aggressive patients was schizophrenia, as already reported in another Italian study by Grassi et al [27]. The same study reported that most violent patients had had previous psychiatric admissions (92%). However, it was shown that no difference in aggressive behaviours emerges when comparing our results from the overall PERSEO population with those reported by Ballerini et al in the subgroup of patients at their first psychiatric admission [4].

Comparing the treatments prescribed during hospital stay and at discharge with those received by the patients before admission, the most commonly prescribed drugs were benzodiazepines, independent from psychiatric diagnosis or presence of aggressive behaviours, at all time points. Benzodiazepine prescription was statistically higher in women than in men at admission, but this difference disappeared during hospitalization and at discharge, with a global increase of use in both genders. Antidepressants, as expected, were most frequently prescribed for depressive episode/status and neurotic disorders. The most relevant change in psychopharmacological therapies observed after admission to an SPDC was the increase in administration of both atypical and conventional antipsychotics. Up to 83% of patients were prescribed neuroleptic drugs, and 27% had a combination of them. These percentages are not in disagreement with those reported by previous Italian surveys on the use of neuroleptics and other psychotropic drugs in Italian mental health services over the last decade, with 84% of neuroleptics prescriptions among psychiatric inpatients (up to 98% among schizophrenics) and 67–75% among outpatients, with 45 and 28% of combination therapies in in- and outpatients being reported, respectively [28-30]. The rates of prescription of conventional and atypical antipsychotics were quite similar at admission and during SPDC stay, whereas atypical antipsychotics were slightly preferred as a discharge prescription (Table 4). In particular, atypical and conventional antipsychotics were equally prescribed for schizophrenia (Figure 1), while atypical drugs were preferred in the other diagnostic groups, such as personality disorders (Figure 1), schizoaffective psychoses or affective psychoses with manic or even depressive episodes (Figure 2). These data confirm the increasing use of atypical antipsychotics reported in several American and Italian pharmaco-epidemiological studies [31-34] over the last decade, even though they also seem to indicate that conventional antipsychotics are not being completely replaced in clinical practice by modern "atypical" antipsychotics, as commented by Gardner et al in a very recent critical overview [34]. Differences in drug prescription observed between males and females reflect the different diagnosis distributions observed. The percentage of patients receiving combination therapies was quite high at all time points, thus confirming the widespread use of polypharmacy, a pattern that seems to have grown considerably over the last three decades [28,30,35], despite remaining controversial. Many new drugs are available nowadays, and physicians might be motivated to consider polypharmacy in an attempt to improve quality of life and increase efficacy, but some authors have underlined that polypharmacy can also increase the risk of adverse effects, drug interactions, non-compliance, and medication errors [36].

If we compare discharge prescriptions in first psychiatric admission (FPA) patients, as analysed in our previous report [4], with non-FPA patients, no significant differences are observed: benzodiazepines (69% of FPA patients; 69% of non-FPA patients), atypical (in 51 and 53% of patients respectively) and conventional antipsychotics (in 38 and 46% of patients respectively) were the most prescribed drugs for both populations, with schizophrenia being the commonest diagnosis in both groups (31% among FPA patients, 35% in non-FPA patients). By contrast, mood stabilizers were given in a significantly lower proportion of FPA cases (21% vs 35% of non-FPA cases; p < 0.001 with Bonferroni correction for five multiple comparisons), whereas a higher though not significant percentage of antidepressants (41%) were prescribed to FPA than to non-FPA patients (34%; p = 0.2 with Bonferroni correction for five multiple comparisons).

An increase in self-referred compliance was observed from admission to SPDC to discharge, indicating that our patient population's subjective view of drug treatment became more positive during hospital stay.

## Conclusion

We are aware that this survey suffers from the limitations of a naturalistic, observational, non-comparative, non-randomised design; however it offers updated information on the clinical characteristics and management of a considerable population of patients admitted to psychiatric emergency structures spread throughout Italy. With regard to medications, our results confirm the widespread use of antipsychotics and the increasing trend in atypical drug prescription, for both psychiatric in- and outpatients. Positive data emerged regarding patients' perception of treatment, a measure that is increasingly considered crucial to improve the use of psychotropic drugs and enhance medication adherence, which might eventually relate to the clinical outcome of the disease [34,35].

## Competing interests

The study was fully sponsored by Eli Lilly Italy. SF, AR and MS are current employees of Eli Lilly Italy.

## Authors' contributions

AB, RB, GB, MC, FM, LM, RR, FR and AS are members of the PERSEO study Advisory Board. They all contributed to the study protocol design, enrolment, and interpretation of analyzed data and to the review of this paper. SF, AR, MS are current employees of Eli Lilly Italy, the study sponsor. They contributed to the interpretation of analyzed data and to the writing and review of this paper. Finally, the members of the PERSEO study group contributed to patient's enrolment.

## Appendix

### The PERSEO study group

The following centers have collaborated on the PERSEO study:

• Barale F, Bonzano A, Scioli R – Neurol. Inst. of Mondino Pavia

• Bellomo A, De Giorgi A, Cammeo C – Osp. Riuniti Hospital Foggia

• Cao A, Zara B – San Francesco Hospital Nuoro

• Conforti I, Chillemi C – Psychiatric Department Parma

• Dagnino L, Ponzoni M – Ospedali Riuniti Hospital Bergamo

• Della Pietra F, Benettazzo M – Azienda USL 16 Hospital Padova

• Esposito V, Sposito M – Psychiatric Department Palermo

• Fato M, Signorello G – Hospital department of Ponente Genova

• Fiorenzoni S, Singali A – Ponte Nuovo Hospital Firenze

• Margari F, Sicolo M – Policlinico Consorziale Hospital Bari

• Martino C, Leria G – Santa Croce Hospital Torino

• Tavolaccini L, Nigro G – Martini Hospita lTorino

• Russo V, La Rovere R – SS. Immacolata Hospital Chieti

• Righi R, Mazzo M – Hospital of Adria Rovigo

• Rocchetti R, De Martiis L – Umberto I Hospital Ancona

• Rodighiero S, Morello M – Hospital of Monselice Padova

• Vescera M, Pisciotti DG – Iannelli Hospital Cosenza

• Villari V, Barzegna G – Molinette S. G. Battista Hospital Torino

• Annicchiarico V, Cosmai MG – Hospital of Venere Bari

• Rossi G, Baraldi EC – Poma Hospital Mantova

• Casacchia M, Ruggiero D – San Salvatore Hospital L'Aquila

• Galimberti P, Fellini FA – Angelucci Hospital Roma

• Francobandiera G – Civil Hospital Sondrio

• Gaspari D, Turati D – SSS. Trinità Hospital Novara

• Matacchieri B, Moscati G – Hospital Taranto

• Mautone A, Casale M – Hospital of Sant'Arsenio Salerno

• Mellado C, Scaramelli B – L. Sacco Hospital Milano

• Filippo A, Miccichè M – Beato Angelo Hospital Cosenza

• Minervini L, Banzato C – Azienda USL 16 Hospital Padova

• Orengo S, Alisio G – San Paolo Hospital Savona

• Picci RL, Venturello S – S. Luigi Gonzaga Hospital Torino

• D'Aloise A, Vaira F – S. Timoteo Hospital Campobasso

• Boccalon RM, Cavrini L – Sant' Anna Hospital Ferrara

• Cogrossi S, Prato K – Osp. Maggiore Hospital Cremona

• Cremonese C, Menardi A – Azienda Hospital Padova

• Parisi M, Mentastro C – Umberto I Hospital Enna

• Prosperini P, Binda V – Magg. della Carità Hospital Novara

• Romano G, Materzanini A – Mellino Mellini Hospital Brescia

• Crudele A, Stella G – Hospital of Barletta Bari

• Petio C, Fuà B – Ottonello Institute Bologna

• Laich L, Miori M – Hospital department of Arco Trento

• Salteri A, Catania G – Vimercate Civil Hospital Milano

• Achena M, Fara FM – Hospital of Sassari Sassari

• Padoani W, Compagno S – Hospital of Conegliano Treviso

• Ballerini A, Pecchioli S, Moretti S – S.M.N. Hospital Firenze

• Bacchi L, Vicari E – Hospital of Partinico Palermo

• Arvizzigno C, Minunni P – F. Iaia Hospital Bari

• Rossi E, Zaiti MF – L. Pierantoni Hospital Forlì Cesena

• Boncompagni G, Selleri MS – O. Malpighi Hospital Bologna

• Minnai GP, Loche AP – San Martino Hospital Oristano

• Russo F, Antonucci A – Nuovo R. Margherita Hospital Roma

• Chiurco L, Amendola R – G. Compagna Hospital Cosenza

• De Giovanni MG, Martano A – V. Fazzi Hospital Lecce

• Borsetti G, Santone G – Umberto I Hospital Ancona

• Pettolino AR, Lisanti F – Umberto I Hospital Foggia

• Parodi A, Ciammella L, Botto G – Villa Scassi Hospital Genova

• Gillotta S, Florio G – Cannizzaro Hospital Catania

• Fiore F, Santangelo E – A. Landolfi Hospital Avellino

• Fucci G, Ricci M – Psychiatric Department Ravenna

• Ciaramella A, Della Porta A – S. Sebastiano M. Hospital Roma

• Sittinieri M, D'Asta L – Paternò Arezzo Hospital Ragusa

• Triolo S, Spatola A – ARNAS Civil Hospital Palermo

• Frediani S, Rossi A, Macchi S, Giovannini L, Germani S, Fabbri L – Eli Lilly Italia, Florence, Italy

• Fiori G (project leader), Sala S (clinical project manager assistant), Sgarbi S (clinical project manager), Simoni L (statistics), Zanoli M (clinical data manager) – MediData Studi e Ricerche s.r.l; c/o Centro Servizi CittàNova Viale Virgilio 54/U, 41100 MODENA, Italy.
